# Primary diffuse leptomeningeal gliomatosis as a rare cause of pain in cervical spine

**DOI:** 10.1186/s12885-016-2224-2

**Published:** 2016-03-04

**Authors:** Štefan Sivák, Ema Kantorová, Egon Kurča, Juraj Marcinek, Pavol Slávik, Jozef Michalik, Vladimír Nosáľ

**Affiliations:** Clinic of Neurology, Jessenius Faculty of Medicine in Martin, Comenius University in Bratislava, Kollárova 2, 03659 Martin, Slovak Republic; Department of Pathological Anatomy, Jessenius Faculty of Medicine in Martin, Comenius University in Bratislava, Kollárova 2, 03659 Martin, Slovak Republic

**Keywords:** Primary diffuse leptomeningeal gliomatosis, Glioblastoma multiforme, Persistent back pain, Cervical spine

## Abstract

**Background:**

Primary diffuse leptomeningeal gliomatosis (PDLG) is a very rare neuro-oncological disease, with only 90 cases of PDLG described in medical literature so far.

**Case presentation:**

We present a case report of a 56-years-old female patient, who was originally hospitalized due to cervical spine pain lasting several months. Despite complex diagnostics and treatment, the neurological state of the patient progressively deteriorated. Patient died 10 months after the first reported symptom. Postmortem pathological findings resulted in the diagnosis of PDLG.

**Conclusions:**

Affection of the cervical spine in early stages of PDLG is rare and has been described in only six patients so far. PDLG is a fatal neuro-oncological disease and it must be kept in mind in the differential diagnosis of persistent back pain syndromes.

## Background

Meningeal (leptomeningeal) gliomatosis is defined as infiltration of subarachnoidal spaces by glial tumor cells. There are two types - primary and secondary meningeal gliomatosis. Secondary leptomeningeal gliomatosis occurs in 8–27 % of patients with primary malignant glioma in the brain or spinal cord. On the other hand, primary diffuse leptomeningeal gliomatosis (PDLG) shows no signs of tumor cells in brain or spinal cord tissue. It is a rare neuro-oncological disease, with only 90 cases of PDLG described in medical literature so far [1, 2].

We present a case report of a female patient with PDLG, who was originally hospitalized due to cervical spine pain lasting several months.

## Case presentation

Our 56-years-old patient (with a history of seropositive rheumatoid arthritis treated by methotrexate since 2005) had been complaining about fluctuating cervical spine pain radiating to occipital region since June 2013. In the beginning of November 2013 the pain worsened and led to 2-week hospitalization with intensive analgesic treatment. On 27/Oct/2013, during ordinary daily activities, she suddenly stopped communicating and looked fixedly in front of her. In hospital, neurological examination revealed perceptive and expressive aphasia as well as palpitation pain in cervical paravertebral muscles with no signs of meningeal irritation. Due to suspected vascular etiology with normal brain CT findings and fulfilled time window criteria, we indicated intravenous thrombolysis and the patient rapidly improved ad integrum. Another possible diagnosis we considered was partial complex epileptic seizure. Psychological examination revealed mildly impaired memory and executive faculties. The other tests performed (internal and ophtalmological examination, EEG, carotid and vertebral artery ultrasound, and CT angiography of intracranial arteries) showed normal findings. Patient was discharged from the hospital with appointments set for further tests of thrombophilic states and MRI of brain and spinal cord.

**Fig. 1:**
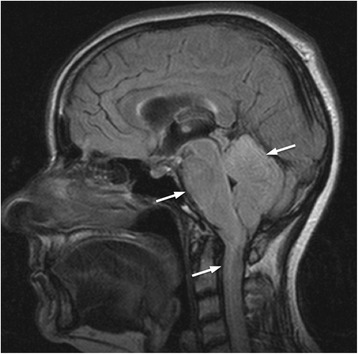
Diffuse T2/FLAIR hyperintensity in cerebellum, brainstem and cervical spinal cord (*arrows*)

**Fig. 2:**
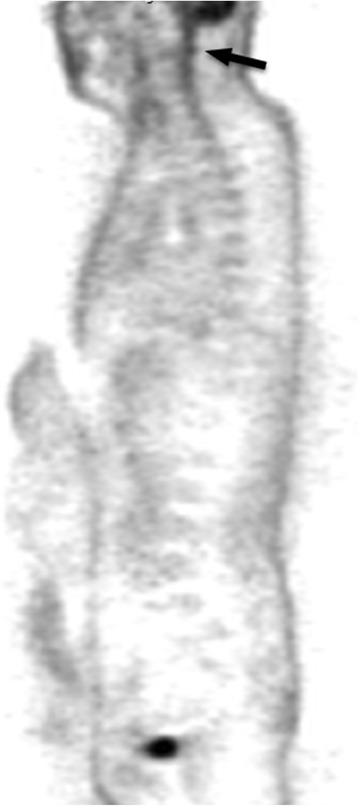
Half-body FDG-PET showing intense uptake along the brainstem, cerebellum, cervical and thoracical spinal cord (*arrow*)

**Fig. 3:**
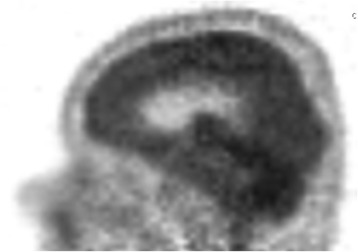
FDG-PET showing increased uptake in infratentorial regions, and partially on brain convexity

On 18/Dec/2013 the patient was again admitted to Neurological clinic due to sudden onset of aphasia with desorientation followed by secondary generalized tonic-clonic epileptic seizure, which improved after symptomatic treatment with diazepam. On neurological examination the patient reported pain in cervical region with no sign of meningeal irritation, had lower limb hyperreflexia, and mild paraparetic-ataxic gait. Brain and spinal cord MRI showed diffuse hyperintensity in hippocampus bilaterally, in brainstem and cerebellum, and in spinal cord grey matter up to segment Th7-8 (Fig. 1). Proton single-voxel MRI spectroscopy revealed no definite pathological metabolic changes in voxel placed in hyperintensive brainstem. There were no signs of cerebrospinal fluid flow obstructions. Cerebrospinal fluid showed massive increase in proteins (12 g/l), increased albumin (4.0 g/l), mild increase in cell count (monocytes 22/3, lymphocytes 5/3) and CD4/8 index (2.94) based on flow cytometry analysis. No oligoclonal bands and erythrocytes were detected. Normal findings were in the following tests: complete blood count, coagulation, basic metabolic panel, oncomarkers (CEA, Ca 72–4, Ca 15–3, Ca 125, Ca 19–9, CYFRA 21–1, SCC antigen), hormons (PRL, FT4, TSH), pathogen tests (lues, borrelia, chlamydia, mycoplasma, EBV, CMV, HSV1, HSV2, VZV, HIV, TBC, toxoplasma, rubeola), antineural IgG antibodies (anti-Ri, Yo, Hu, PNMA2, CV2, amphiphysin), and wide spectrum of autoantibodies (anti- nRNP, anti- Sm, anti- Ro-52, anti- SS-A, anti- SS-B, anti- Scl-70, anti- PM-Scl, anti- Jo-1, anti- CENP B, anti- dsDNA, anti- histone, anti- nucleosome, anti- ribosomal P protein, anti- mitochondrial M2 subtype, anti-tissue transglutaminase, anti-gliadin, anti-EMA, anti- cardiolipin). FDG - PET examination showed increased metabolism regions in meninges in several places: in cervical and thoracical spinal cord, in infratentorial regions, and partially on brain convexity (Figs. 2 and 3). The most probable diagnosis was autoimunne meningoencephalitis with secondary epilepsy. During the long-term hospitalization patient was treated with methylprednisolon (overall dose 5 g), plasmapheresis (3 times), intravenous immunoglobins (0.4 g/kg/day for 5 days), and empirically with acyclovir and ceftriaxone.

Despite extensive treatment the patient progressivelly deteriorated, she developed central lesion of right facial nerve, left upper limb dysmetria, and mild spastic lower-limb paraparesis. She suffered repeated epileptic seizures despite combination of antiepileptic treatment with carbamazepine (2 × 300 mg), levetiracetam (2 × 1000 mg), and lamotrigine (2 × 25 mg). At the beginning of February 2014 the patient began to be somnolent and later semicomatose. Brain MRI showed hydrocephalus with enlargement of all four ventricles, and there was bilateral optic papilla oedema with haemorrhage. External lumbar drainage led to mild improvement - the patient was somnolent with meningeal irritation, she was blind with organic psychosyndrome. Cerebrospinal fluid again showed massive hyperproteinorrhachia (12.9 g/l), pleocytosis (monocytes 52/3, lymphocytes 2/3). Interictal EEG showed mildly abnormal background activity with mixed (slow and epileptiform) regional abnormality in bilateral temporal regions. At the end of February MRI showed mild decrease in intensity od signal in hippocampus, brainstem, cerebellum, and in spinal cord, and remaining hydrocephalus. Meninges were enhanced in frontoparietal regions with MRI contrast aplication. Despite the treatment and complex care, neurological state progressively deteriorated. During April patient developed severe bronchopneumonia with sepsis, left subclavian vein thrombosis followed by multiorgan failure. Patient died on 26/Apr/2014, approximately 10 months after the first report of cervical spine pain.

**Fig. 4:**
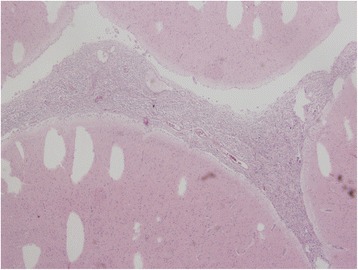
Diffuse infiltration of leptomeninges by tumorous cells, without formation of macroscopically identifiable mass, without infiltration of brain parenchyme. Hematoxylin-eosin stain (20x)

**Fig. 5:**
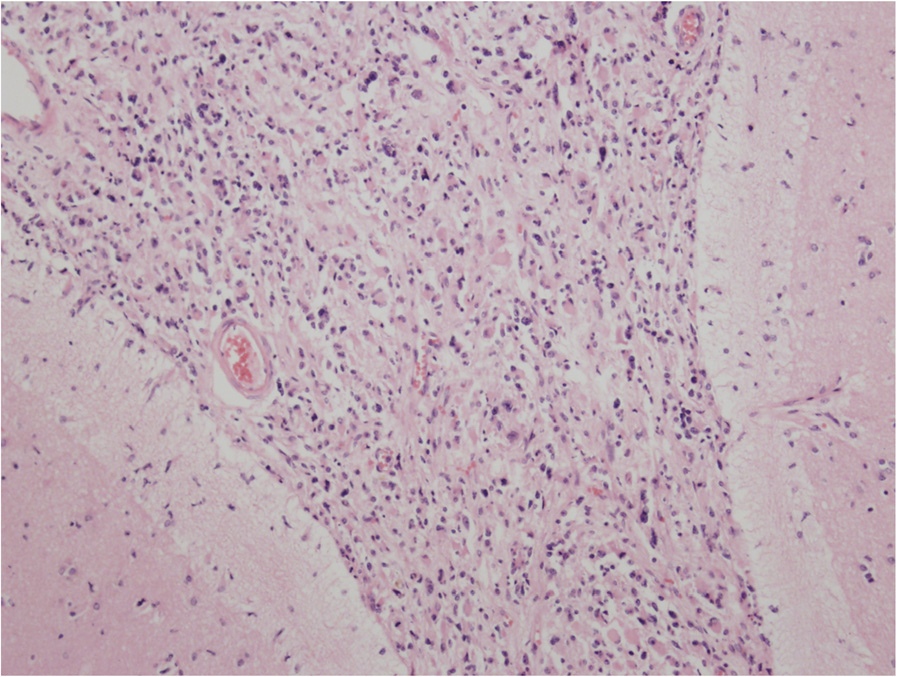
Tumorous mass composed of pleomorphous cells with abundant eosinophilic cystoplasm and variable sized nuclei with pale chromatin and small nucleoli. Tumorous cells with large, irregular, hyperchromatic nuclei and multinucleated cells are intermingled. Hematoxylin-eosin stain (100x)

**Fig. 6:**
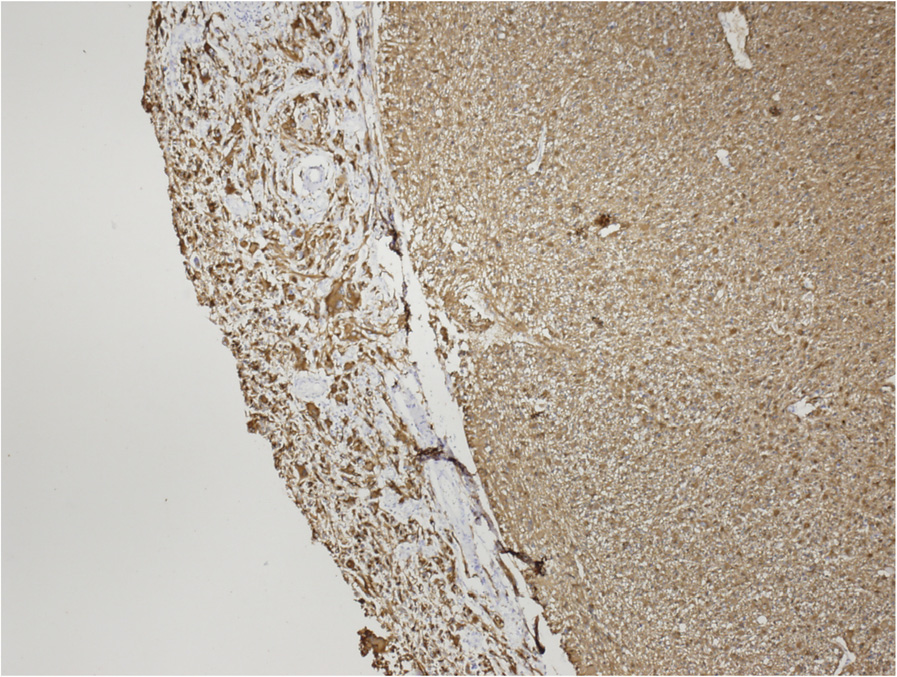
Diffuse cytoplasmatic immunohistochemical positivity of glial fibrillary acidic proteine (GFAP) proves the glial/astrocytic differentiation of tumorous burden (DAKO antibody 40x)

**Fig. 7:**
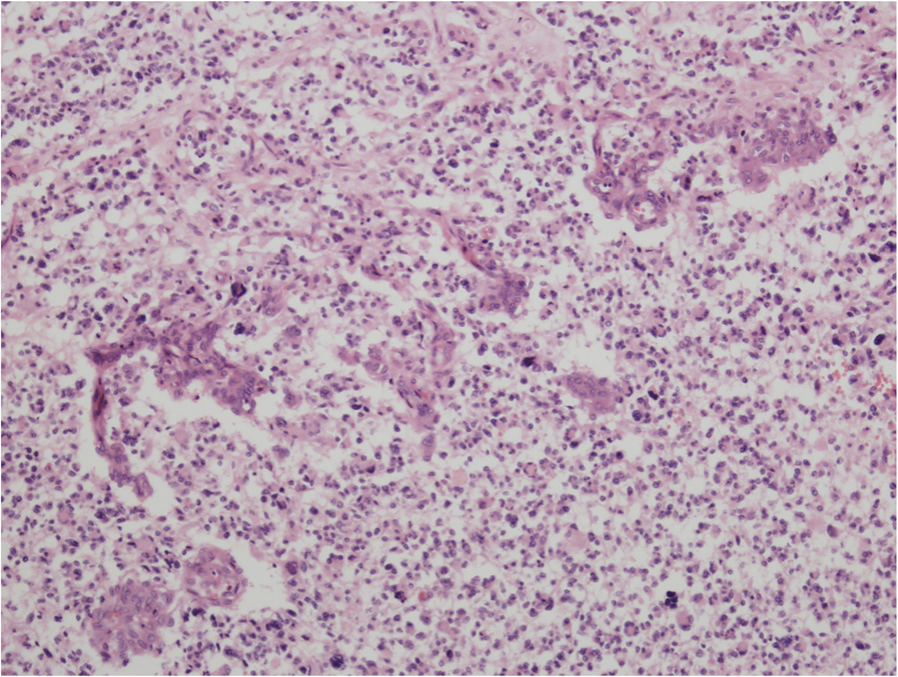
Localised vascular proliferation including s.c. glomeruloid formations characteristic for glioblastomas. Hematoxylin-eosin stain (100x)

The autopsy verified pathology in leptomeninges of medulla oblongata, pons and basal parts of hemispheres. This was extensive tumour infiltration mainly consisting of medium-sized cells with eosinophilic cytoplasm and oval nuclei with granular chromatin, as well as a number of big, multinuclear cells, and cells with large, dense, bizarre nuclei and numerous mitoses including atypical mitoses. Foci of vessel proliferation were also present. Imunohistochemical staining revealed glial fibrillary acid protein (GFAP), S-100 protein, epithelial membrane antigen (EMA), but no signs of CD68 antigen. Biopsy resulted in the diagnosis of glioblastoma multiforme. Despite sampling of whole brain and spinal cord tissue, no similar tumorous lesion was found. These findings match criteria for PDLG (Figs. 4, 5, 6 and 7).

## Conclusions

Since the first description of PDLG in 1923, less than 90 cases have been described so far [1–3]. It is supposed that heterotopic location of glial cells in leptomeninges most probably occurs during embryogenesis. In general population it is present in about 1 % of autopsies and up to 25 % of autopsies of patients with CNS malformations [4]. The age ranges between 1 and 84, with average survival of 4 months [5, 6].

CSF fluid obstruction can be caused by increased amount of proteins in CSF, or tumour-caused subarachnoidal bleeding, infiltration of basal subarachnoidal cisterns, or venous sinuses compression [7]. In our 56-year-old patient hydrocephalus was developed at a later stage of the disease (7 months after first cervical pain). We consider infiltration of subarachnoidal spaces in basal brain regions together with CSF resorbtion impairment due to hyperproteinorrhachia (up to 12.9 g/l) to be the main cause of hydrocephalus.

Other typical early signs of the disease are cranial neuropathies (in 56 % of patients), meningeal irritation (44 %) and epileptic seizures (44 %) [8, 9]. In our patient meningeal irritation occured at a later stage (7–8 months after the first symptoms). The first symptom was treatment-resistant neck pain. It was followed by repeated partial complex epileptic seizures (the first was considered as ischemic stroke with aphasia) 5 months later, and by development of focal spinal neurological deficit (paraparetic-ataxic gait with hyperreflexion in lower limbs). Affection of cervical spine in early stages of PDLG is rare and has been described in only six patients [6, 7, 9–12]. The most common early-stages PDLG location is supratentorial [8, 9].

We explain our patient’s diffuse T2/FLAIR hyperintensity in temporal lobes, brainstem, cerebellum, and in proximal spinal cord to be intraparenchymal oedema developed due to microcirculation impairment caused by tumor infiltration of subarachnoidal spaces. Similar findings were described by other authors [9, 13]. We suppose this to be the cause of our patient’s early neurological symptoms.

Brain MRI in the begining of the disease usually shows normal findings. Later on hydrocephalus is developed, and diffuse thickening and contrast enhancement of the leptomeninges occurs [2, 14]. CSF examination typically shows increased CSF pressure, hyperproteinorrhachia (up to 12.9 g/l in our patient) and lymphocytic pleocytosis with normal or lowered glucose amount can be present [15, 16]. Unlike leptomeningeal carcinomatosis and secondary meningeal gliomatosis, cytologic CSF examination is usually not sensitive enough in PDLG [17, 18]. It is usually a biopsy from the site of leptomeningeal thickening that confirms the diagnosis [14].

PDLG is included in the large differential diagnosis of subacute and chronic meningitis/meningoencephalitis and meningeal tumours [19, 20]. Morphological subtypes of PDLG are glioblastoma multiforme, high-grade astrocytoma, oligodendroglioma, gliosarcoma, and occassionally pilocytic astrocytoma. Other tumours primarily affecting leptomeninges are ependymoblastoma, primitive neuroectodermal tumour, melanocytoma, and lymphomas [14]. There is no standard therapy of the disease at present [9]. It is important to treat the symptoms (e.g. analgesics, antiepileptic therapy, shunt implantation). In early diagnosis of low-grade tumours, case reports suggest the efficacy of chemotherapy (systemic, intrathecal, and intraventricular), and radiotherapy (brain, spinal cord) in prolonged survival of patients [5, 6, 21].

PDLG is a very rare, fatal neuro-oncological disease and it must be kept in mind in the differential diagnosis of persistent back pain syndromes.

### Consent

Written informed consent was obtained from the patient daughter for publication of this Case report and any accompanying images. A copy of the written consent is available for review by the Editor of this journal.

## Abbreviations

CSF: cerebrospinal fluid
CT: computed tomography
EEG: electroencephalography
EMA: epithelial membrane antigen
FDG: PET - fluorodeoxyglucose positron emission tomography
GFAP: glial fibrillary acid protein
MRI: magnetic resonance imaging
PDLG: primary diffuse leptomeningeal gliomatosis
